# Quantitative proteomics and metabolomics analysis reveals the response mechanism of alfalfa (*Medicago sativa* L.) to *o-*coumaric acid stress

**DOI:** 10.1371/journal.pone.0295592

**Published:** 2023-12-08

**Authors:** Xiaoyang Xu, Feilong Geng, Weihong Sun

**Affiliations:** 1 School of Economics and Management, Shandong Agricultural University, Shandong, China; 2 College of Grassland Science and Technology, China Agricultural University, Beijing, China; University of Brescia: Universita degli Studi di Brescia, ITALY

## Abstract

*O-*coumaric acid (OCA), as a significant phenolic allelochemical found in hairy vetch (*Vicia villosa* Roth.), that can hinder the growth of alfalfa (*Medicago sativa* L.), particularly the growth of alfalfa roots. Nonetheless, the mechanism by which OCA inhibits alfalfa root growth remains unclear. In this study, a liquid chromatography tandem mass spectrometry (LC-MS/MS)-based quantitative proteomics analysis was carried out to identify differentially accumulated proteins (DAPs) under OCA treatment. The findings indicated that 680 proteins were DAPs in comparison to the control group. Of those, 333 proteins were up-regulated while 347 proteins were down-regulated. The enrichment analysis unveiled the significance of these DAPs in multiple biological and molecular processes, particularly in ribosome, phenylpropanoid biosynthesis, glutathione metabolism, glycolysis/gluconeogenesis and flavonoid biosynthesis. The majority of DAPs reside in the cytoplasm (36.62%), nucleus (20.59%) and extracellular space (14.12%). In addition, phenylalanine deaminase was identified as a potential chemical-induced regulation target associated with plant lignin formation. DAPs were mainly enriched in flavonoid biosynthesis pathways, which were related to plant root size. Using the UPLC-ESI-MS/MS technique and database, a total of 87 flavonoid metabolites were discovered. The metabolites were predominantly enriched for biosynthesizing naringenin chalcone, which was linked to plant lignin formation, aligning with the enrichment outcomes of DAPs. Consequently, it was deduced that OCA impacted the structure of cell walls by mediating the synthesis of lignin in alfalfa roots, subsequently inducing wilt. Furthermore, a range of proteins have been identified as potential candidates for the breeding of alfalfa strains with enhanced stress tolerance.

## Introduction

Alfalfa (*Medicago sativa* L.) is a high-quality forage crop, rich in protein [1]. However, its growth performance gradually declines with age due to its developed root system. The taproot of alfalfa can penetrate deep into the soil, reaching up to 10 m or even 30 m [2], which makes it difficult to remove it by cutting or other means. After crop rotation, alfalfa may frequently be found in fields used for grain crops, which can have a notable effect on the growth and yield of ensuing crops [3]. Therefore, we use the allelochemicals discharged by hairy vetch (*Vicia villosa* Roth.) to eliminate any remaining alfalfa roots. This leguminous plant, hairy vetch, effectively hinders weed proliferation [4]. The growth of weeds is inhibited by the chemicals contained in this plant, and allelopathy is believed to play a significant role in weed control [5].

The study has demonstrated that *o-*coumaric acid (OCA) (S1A Fig) is a significant allelochemical present in the stems and leaves of hairy vetch. OCA is a white needle crystal, easily soluble in alcohol and hot water, with a melting point of 214 °C. Its application significantly inhibits the growth of alfalfa roots [6]. Numerous studies have explored the inhibitory mechanism of allelopathy. The inhibition mechanism could arise from alterations in plant cell structure, which may affect the lipidome, membrane integrity, and photosynthesis [7]. Moreover, imbalances in theantioxidant system, the disruption of enzymatic activity, the influence of nutrient absorption by plant roots or the impace on nucleic acid [8], disrupt hormine balance in seeds.

OCA is a phenolic compound, and Zhou *et al* conducted a study that revealed phenolic compounds such as 3-(2-hydroxyphenyl) propyl methyl malonate (S1B Fig), 3-(2-hydroxyphenyl)-1-propanol (S1C Fig) and OCA remarkably inhibit *A*. *thaliana* (*Arabidopsis thaliana* (L.) Heynh) seed germination at a concentration of 1.0 mM [9]. Moreover, OCA was found to have noticeable inhibitory effects on *A*. *thaliana* seedling growth when cultivated in a soil medium [10].

In recent years, proteomics and metabolomics have emerged as popular techniques for identifying proteins and metabolites linked to biotic and abiotic stress responses in plants [11]. The proper regulation of protein and metabolite levels is crucial for responding to biotic and abiotic stresses as most gene functions rely on the translation of transcripts into these molecules. Nevertheless, there is a scarcity of systematic comparisons and analyses of protein and metabolite level response mechanisms in alfalfa to OCA stress. Quantitative proteomics and metabolomics analyses were conducted on alfalfa root samples treated with OCA using LC-MS/MS and UPLC-ESI-MS/MS techniques. The results pinpointed the significant differences and identified the principal proteins and metabolites triggered by OCA and the related pathways. Not only does it reveal the mechanism of OCA in plants, but it also contributes critical date for forthcoming studies on the response genes of OCA and the exploration of OCA ability to remove perennial alfalfa roots.

## Materials and methods

### Experimental materials

OCA (with a purity of 95%) was procurd from Beijing Bailingwei Technology Co., Ltd (Beijing, China) for the seedling growth experiment. The alfalfa variety used is Suntory (*Medicagosativa* L.cv.Sanditi), which was originally from the Netherlands. It was introduced into China in 1997 by the Green Group China Representative Office, having been purchased from Beijing Zhengdao Seed Industry Co., Ltd (Beijing, China).

Fifty seeds from the aforementioned species were placed equidistantly in Petri dishes (diameter = 90 mm) lined with two filter paper layers. Each dish was then treated with 10 mL of one of eight OCA solutions at varying concentrations (0, 0.05, 0.1, 0.2, 0.4, 0.8, 1.6, 3.2 g/L) independently. Ten samples were subsequently extracted to measure the hypocotyl length on the 7th day. The pre-germinated seeds were grown in Hoagland solution. Afterwards, varying doses of OCA (0, 0.5, 1, 1.5, 2, 2.5 g/L) were introduced. Each treatment was replicated three times. The length of the roots was measured after 7 d, with ten samples taken for each group.

The alfalfa seeds were initially pre-germinated. Next, six uniform alfalfa seedlings in the 2-leaf growth stage were carefully transplanted into a polystyrene foam board having six perforations (16 × 9 cm), and stabilized using a tampon that was inserted into each well. Therafter, the polystyrene foam board with seedlings was placed in a plastic pot (length 16 cm, width 9 cm, height 6 cm), which contained 0.5 L of Hoagland solution and 0.5 g/L of OCA solution. Hoagland solution without induction was used as the control in the experiment. The plastic pots were placed in an incubator at a temperature of 30 ± 2 °C under a light intensity of 70 μmol/(m^2^s) for 12 h (8:00–20:00 h) and this experiment was repeated three times. After 7 d of treatment, all alfalfa roots were harvested.

#### Sample preparation and extraction

The freeze-dried root was ground using a micropipette grinder (Tissuelyser-II, Leimeng Biology Science and Technology Co., Ltd, Shanghai, China) to obtain a fine cell powder, which was subsequently transferred into a 5-millilitre centrifuge tube. Four volumes of lysis buffer (8 M urea, 1% Triton-100, 10 mM dithiothreitol, and 1% Protease Inhibitor Cocktail) were subsequently added to the cell powder, followed by three rounds of sonication on ice using a high-intensity ultrasonic processor (Scientz, Shanghai Jingxin Industry Development Co., Ltd, Shanghai, China). The KH30R model if freezing centrifuge (Hainan Kaida Scientific Instrument Co., Ltd, Hainan, China) was employed in the processed. Protein precipitation was achieved by using cold 20% TCA (trichloroacetic acid) for 2 h at -20 °C. The residual debris was eliminated by subjecting it to centrifugation at 20,000 g at 4 °C for 10 min. Centrifugation was carried out at 12,000 g at 4 °C for 10 min, following which, the supernatant was discarded. The residual precipitate was then cleaned thrice with cold acetone. The protein was dissolved again in 8 M urea, and its concentration was measured by using the BCA kit (Elabscience Biotechnology Co.,Ltd, Wuhan, China) following the manufacturer’s instructions.

#### Trypsin digestion

For the protein digestion process, a 5 mM dithiothreitol reduction was performed on the solution at a temperature of 56 °C and left for 30 min. An 11 mM iodoacetamide alkylation step in the dark at room temperature for 15 min ensued. Subsequently, a dilution process took place by adding 100 mM TEAB (Tetraethyl-ammonium bromide) until the urea concentration was less than 2M. Afterwards, the addition of trypsin followed, using a trypsin-to-protein mass ratio of 1:50 for overnight digestion and 1:100 for a second 4-h digestion.

#### LC-MS/MS analysis

The tryptic peptides were dissolved in 0.1% formic acid (solvent A) before being loaded directly onto a reversed-phase analytical column, which was 15 cm in length and had an inner diameter of 75 μm. The technical term abbreviations used have been clearly explained for clarity. The gradient consisted of a rise from 6% to 23% solvent B (0.1% formic acid in 98% acetonitrile) that occurred over a period of 26 min. This was followed by an increase from 23% to 35% in 8 min. The gradient then climbed constant at 80% for the last 3 min. All these step were done at a constant flow rate of 400 nL/min using an EASY-nLC 1000 UPLC system (Hunan Dongli Intelligent Technology Co., Ltd, Hunan, China).

The peptides underwent NSI source processing, followed by tandem mass spectrometry (MS/MS) using Q Exactive^™^ Plus (Thermo), which was coupled online to the UPLC. An electrospray voltage applied of 2.0 kV was applied. The mass-to-charge ratio (m/z) scan range was 350 to 1800 m/z for full scanning, and intact peptides were detected in the Orbitrap with a resolution of 70,000. Peptides were then chosen for MS/MS using the NCE setting at 28, and the fragments were detected in the Orbitrap with a resolution of 17,500. A data-dependent method was utilized which alternated between one MS scan and 20 MS/MS scans, with a dynamic exclusion of 15.0 s. The automatic gain control (AGC) was set at 5E4, with a fixed first mass of 100 m/z.

#### Protein identification and quantitation

The MS/MS data obtained were analysed utilising Maxquant (v1.6.6.0). The retrieval parameters were set such that Uniprot Medicago _ Truncatula (containing 57065 sequences) was used as the database. Moreover, an anti-database was incorporated to compute the false positive rate (FDR) resulting from random matches, the common pollution database was also employed to eliminate the impact of polluted to proteins in the identification outcomes. Enzyme digestion was carried out using trypsin/p, with a determined number of 2 missing cleavage sites. The tolerance of mass error for primary parent ions was set to 40 ppm for both the First and Main searches, while the tolerance of mass error for secondary fragment ions was set to 0.04 Da. Fixed modification for cysteine was set as alkylation and the variable modification was oxidation of methionine and acetylation of protein N-terminal. The FDR for protein identification and PSM identification was set at 1%.

#### Functional analysis of DAPs

We performed functional and enrichment analysis of DAPs using GO (http://www.geneontology.org) and KEGG (http://www.genome.jp/kegg/). We considered the GO pathways with a significance level of *P* < 0.05 and KEGG pathways with a significance level of *P* < 0.01 as the significant enrichment pathways.

#### UPLC-ESI-MS/MS analysis

Samples were obtained from alfalfa roots, following the aforementioned procedure. Subsequently, an approximately 0.2 g sample was separately measured and ground in liquid nitrogen using the micropipette grinder (Tissuelyser-II, Leimeng Biology Science and Technology Co., Ltd, Shanghai, China). The extracted samples were processed employing previously described techniques. Flavonoid metabolites were quantified via ultra high performance liquid chromatography-electrospray tandem quadrupole mass spectrometry (UPLC-ESI-MS/MS) (Waters, Beijing Jing ke Rui da technology co., ltd, Beijing, China) with an external standard. The samples were filtered through a 0.22 μm membrane syringe filter and then injected onto a C18 column (250 × 4.6 mm; 5 μM; Knauer, Sweden). The mobile phase consisited of ultra-pure water with the addition of 0.04% acetic acid. The organic phase was made up of acetonitrile, also with 0.04% acetic acid added. Chromatography proceeded by eluting using a water and acetonitrile mixture at a flow rate of 0.4 mL/min. The column was kept at a temperature of 40 °C, with a sample size of 5 μL. The ratio of water to acetonitrile were 95:5 V/V at 0 min, 5:95 V/V at 11.0 min, 5:95 V/V at 12 min, 95:5 V/V at 12 min, and 95:5 V/V at 15 min, respectively. The experiments were replicated three times.

#### Quantitative and qualitative analysis of differentiated flavonoids metabolites

The qualitative analysis of the primary and secondary spectrum data of mass spectrometry was undertaken, based on the public metabolite information database and our own MWDB (metware database). During the analysis, certain substances were qualitatively assessed, and isotope signals, repeated signals containing K^+^, Na^+^ and NH4^+^ and repeated signals of fragment ions which are othersubstances with larger molecular weight were excluded. Metabolite structure analysis is conductedthrough the use of MassBank (http://www.massbank.jp/), knapsack (http://kanaya.naist.jp/KNApSAcK/), HMDB (http://www.hmdb.ca/), MoTo DB (http://www.ab.wur.nl/moto/) and Metlin (http://metlin.scripps.edu/index.php) databases. The quantification of metabolites has been achieved using the multiple reaction monitoring (MRM) analysis of triple quadrupole mass spectrometry. In MRM mode, the four-stage rod first screened the precursor ions (parent ions) of the target substance undergo screening by the for-stage rod, which excludes ions corresponding to other molecular weight substances to preliminarily eliminate interference preliminarily. The collision chamber induced precursor ion ionization, leading to the formation of numerous fragment ions. The fragment ions were filtered using triple quadrupole to select the necessary characteristic fragment ion, thereby eliminating any interference from non-target ions and enhancing the accuracy and repeatability of quantification. Upon obtaining the data of metabolite mass spectrometry analysis data from various samples, the peak area of all the mass spectrometry peaks was integrated, and the peak of the same metabolite in different samples was integrated and corrected.

#### Statistical analysis

Statistical significance was determined through the use of one-way ANOVA followed by Tukey’s test using SPSS25.0 for all biochemical assays. Graphical and statistical significance representations of significance, presented as mean ± SEM, were created using by MS-Excel 2019.

## Results and discussion

### Effect of OCA on hypocotyl and root length

**Fig 1:**
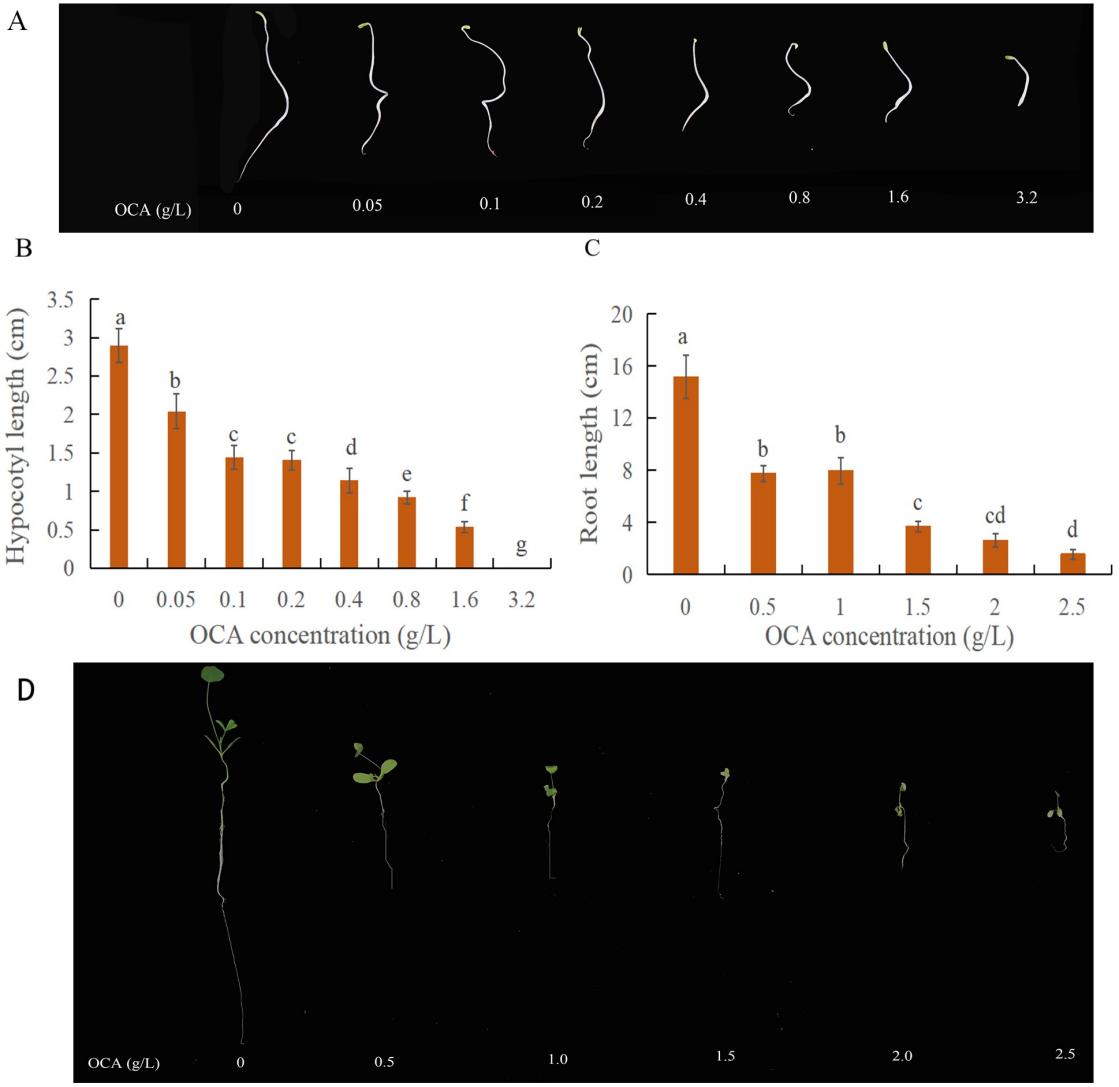
Effect of OCA on the length of hypocotyl and root. (A, B) The length of hypocotyl during alfalfa seed germination. (C, D) The length of the root during alfalfa seedling growth. Different lower-case letters in columns indicate significant differences among treatments of alfalfa at *P* < 0.05 (LSD test).

When the OCA was less than 0.1 g/L, the inhibitory effect on alfalfa hypocotyl was not significant. However, with the increase of OCA concentration, the inhibitory effect gradually strengthened. At a substance concentration of 3.2 g/L, the alfalfa hypocotyl was only 2.16 ± 0.64 cm with the growth inhibition ratio of 76.88%, whereas it was 7.47 ± 0.16 cm under control conditions. The hypocotyl was fully suppressed when exposed to substance concentrations of 3.2 g/L during seed germination. Varying degrees of OCA concentrations notably curtailed the length of alfalfa roots, with an incremental intensification of suppression witnessed with each concentration increase. The root length of alfalfa was 1.54 ± 0.39 cm at a substance concentration of 2.5 g/L, with the growth inhibition ratio of 89.83% in contrast to a root length of 15.16 ± 1.69 cm in the control condition (Fig 1).

### Quantitative validation of proteome quality and protein identification

The aim was to identify the DAPs in the roots of alfalfa treated with 0.5 g/L OCA solution for 7 d and their control (without OCA solution). A total of 274092 spectra and 37776 peptides were detected. Among them, 181991 effective spectra were obtained with a 66.4% utilization rate. The number of specific peptides among the identified peptides was 33716.0, and a total of 6386.0 proteins were identified within them. After removing any repetitive or unqualified proteins with only one unique peptide, 5005 proteins were identified (Table 1).

**Table 1:** Search and analysis of spectrum database combined with liquid chromatography-tandem mass spectrometry (LC-MS/MS).

Total spectrum	Matched spectrum	Peptides	Unique peptides	Identified proteins	Quantifiable proteins
274092.0	181991 (66.4%)	37776.0	33716.0	6386.0	5005.0

### Dynamic proteome profiling of alfalfa root under OCA stress

All 5005 proteins were identified in all experimental groups and were used in quantification for further comparisons (S2 Fig). Applying Duncan variance analysis with a *P* < 0.05, *FC* = 1.5 as the threshold, 680 of the 5005 quantified proteins were identified as DAPs under OCA stress. Of those, 333 DAPs were up-regulated and 347 DAPs were down-regulated.

### Enrichment analysis of DAPs

**Fig 2:**
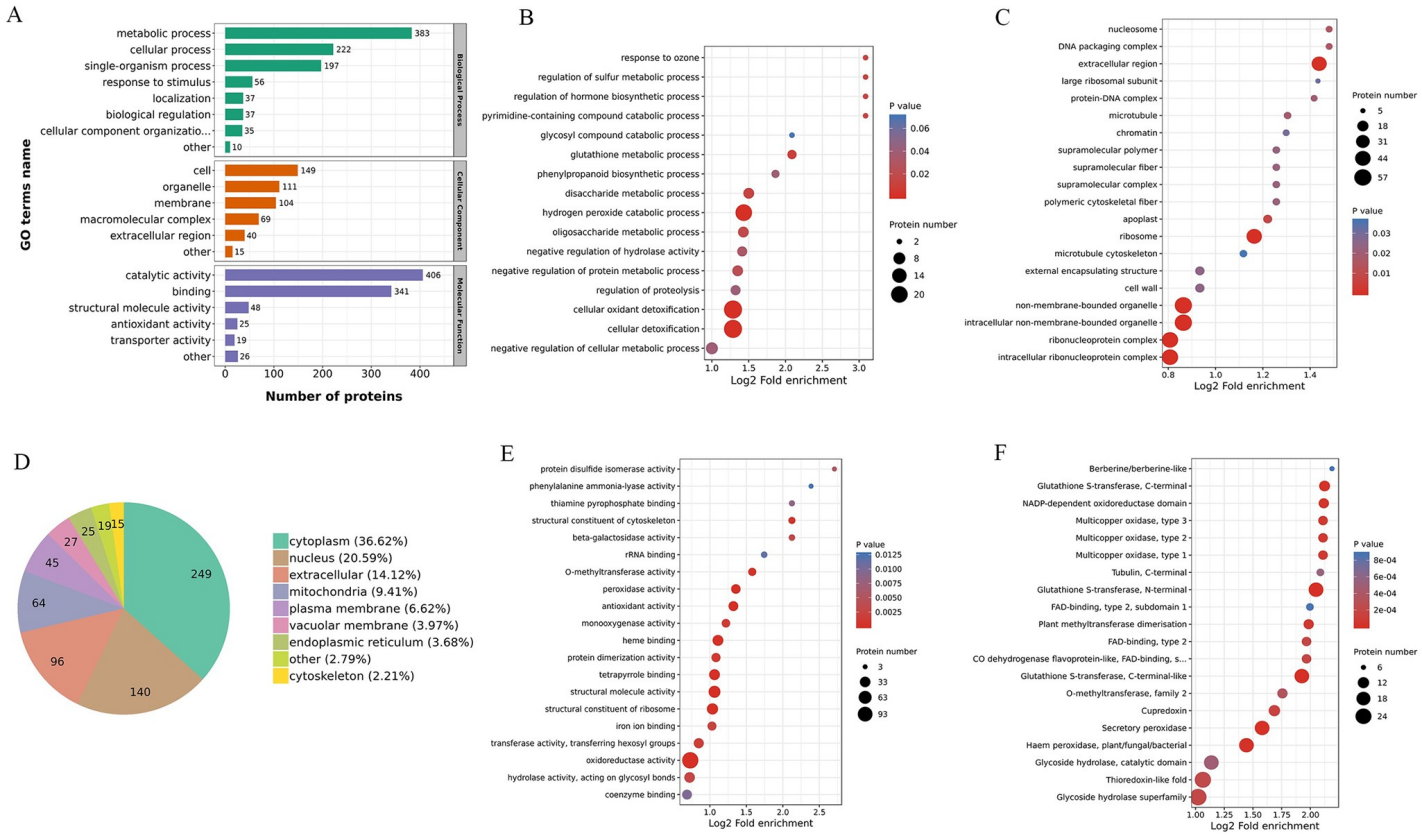
GO annotation of identified DAPs. (A) The identified DAPs were enriched in biological process, cellular component and molecular function. (B) The identified DAPs were enriched biological process. (C) The identified DAPs were enriched cellular component. (D) The prediction of subcellular localization of identified DAPs. (E) The identified DAPs were enriched molecular function. (F) The identified DAPs were enriched protein domain.

To determine the functional groups of DAPs in alfalfa roots under OCA stress, we conducted GO analysis as shown in Fig 2. Our results indicated that DAPs were primarily enriched in the metabolic processes (383), cellular processes (222) and single−organism processes (197). Among the cell components, DAPs were chiefly enriched in cells (149), organelles (111) and membranes (104). In terms of molecular function, DAPs demonstrated significant enrichment in catalytic activity (406) and binding (341) (Fig 2A). To delve deeper into the effect of OCA on the alfalfa root across different biological processes and cell structures, we conducted an in-depth analysis of the relevant biological functions and pathways of DAPs with OCA lack/supplement treatment in alfalfa roots. The findings indicate that the DAPs were significantly grouped under 16 biological processes linked with GO terms (*P* < 0.05), namely ‘cellular detoxification’, ‘cellular oxidant detoxification’, and ‘hydrogen peroxide catabolic process’ (Fig 2B). Among the GO terms associated with cellular components, ‘ribonucleoprotein complex’, ‘intracellular non-membrane-bounded organelle’, ‘non-membrane-bounded organelle’, ‘ribosome’ and ‘extracellular region’ were found to be highly enriched, as shown in Fig 2C. The probability of DAPs residing in the cytoplasm, nucleusand extracellular was 36.62%, 20.59% and 14.12%, respectively (Fig 2D). There were 20 GO terms associated with molecular function, including ‘oxidoreductase activity’, ‘structural molecule activity’, ‘structural constituent of ribosome’, ‘tetrapyrrole binding’, ‘heme binding’, ‘antioxidant activity’ and ‘peroxidase activity’ (Fig 2E). There were 20 protein domain-associated GO terms were identified, including the Glycoside hydrolase superfamily, Thioredoxin-like fold, Haem peroxidase, Secretory peroxidase, Glutathione *S*−transferase, *C−*terminal-like, *N−*terminal Glutathione *S−*transferase (Fig 2F).

### KEGG analysis of DAPs

**Fig 3:**
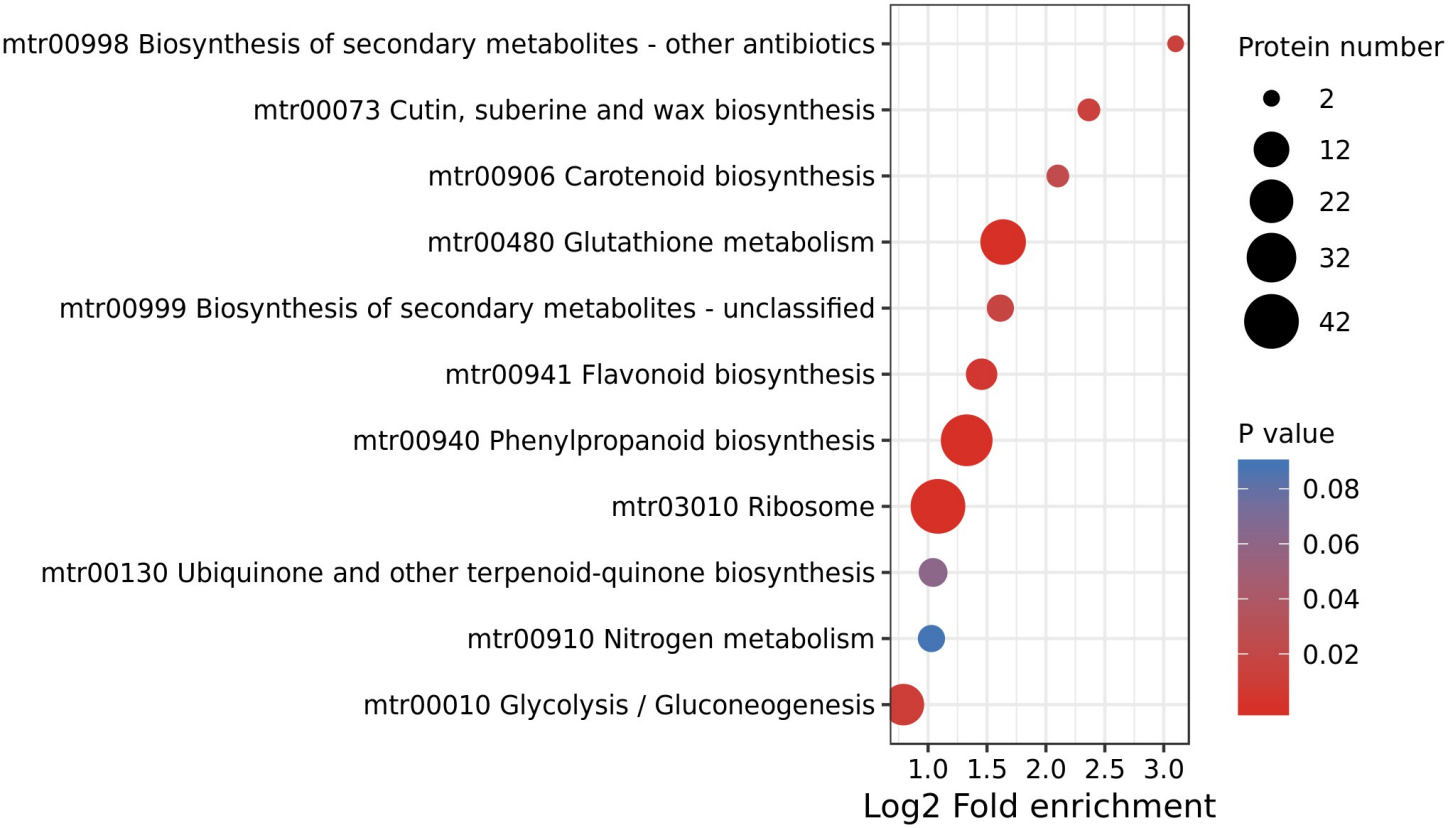
KEGG annotation of identified DAPs.

We also conducted KEGG analysis to further explore the molecular functions of DAPs identified in this study, the highly significant enriched DAPs (*P* < 0.01) were in four pathways: ‘Ribosome’, ‘Phenylpropanoid biosynthesis’, ‘Glutathione metabolism’, ‘Glycolysis/Gluconeogenesis’ and ‘Flavonoid biosynthesis’ (Fig 3).

### Analysis of the identified DAPs enrichment in KEGG pathways

To investigate further the impact of OCA on alfalfa roots in the pathways of glycolysis/gluconeogenesis, glutathione metabolism, phenylpropanoid biosynthesis and flavonoid biosynthesis, we conducted an in-depth analysis of the identified DAPs enrichment in these processes. Our findings reveal that the glycolysis/gluconeogenesis pathway, experienced a down-regulation of aldose 1-epimerase, while the other identified DAPs, 6-phosphofructokinase and fructose-bisphosphate aldolase class I, were up-regulated. Glyceraldehyde-3-phosphate dehydrogenase (NADP+), enolase, phosphoenolpyruvate carboxykinase (ATP), pyruvate kinase, pyruvate decarboxylase, l-lactate dehydrogenase, and alcohol dehydrogenase were all involved in sugar metabolism. Glyceraldehyde-3-phosphate dehydrogenase (S1D Fig) oxidatively phosphorylates glyceraldehyde-3-phosphate, produce 1, 3-diphosphoglyceric acid (S1E Fig), which serves as the central link in this process. When alfalfa roots were exposed to OCA-induced stress, the glycolysis/gluconeogenesis pathway facilitated energy production for the growth of alfalfa roots (Table 2).

**Table 2:** Main enrichment pathways of identified DAPs.

KEGG pathway	Protein	P value	Regulated Type
Glycolysis/Gluconeogenesis	Aldo/keto reductase family oxidoreductase	0.00004	Up
	Aldehyde dehydrogenase	0.00008	Up
	Pyruvate kinase	0.00010	Up
	Phosphopyruvate hydratase	0.00011	Up
	NADP-dependent glyceraldehyde-3-phosphate dehydrogenase	0.00018	Up
	Fructose-bisphosphate aldolase	0.00020	Up
	Alcohol dehydrogenase 1	0.00023	Up
	ATP-dependent 6-phosphofructokinase	0.00040	Up
	Pyruvate decarboxylase	0.00048	Up
	ATP-dependent 6-phosphofructokinase	0.00054	Up
	Pyruvate kinase	0.00080	Up
	Pyruvate kinase	0.00082	Up
	L-lactate dehydrogenase	0.00170	Up
	Putative pyruvate decarboxylase	0.00171	Up
	Phosphoenolpyruvate carboxykinase [ATP] protein	0.00176	Up
	Putative alcohol dehydrogenase	0.00296	Up
	Zinc-binding alcohol dehydrogenase family protein	0.00342	Up
	Aldose 1-epimerase	0.00690	Down
	Pyruvate decarboxylase	0.01380	Up
Glutathione metabolism	Glucose-6-phosphate 1-dehydrogenase	0.00002	Up
	Glutathione *S-*transferase, amino-terminal domain protein	0.00004	Up
	Glutamate—cysteine ligase	0.00033	Up
	Glutathione reductase	0.00164	Up
	Glutathione *S-*transferase tau	0.00258	Up
	Glutathione *S-*transferase	0.00316	Up
	Glutathione peroxidase	0.00478	Up
Phenylpropanoid biosynthesis	Peroxidase	0.00002	Up
	Phenylalanine ammonia-lyase	0.00066	Up
	Caffeic acid *O*-methyltransferase	0.00068	Up
	Glycoside hydrolase family 1 protein	0.00306	Down
	4-coumarate:CoA ligase-like protein	0.00314	Up
	Beta-glucosidase G1	0.00374	Up
Flavonoid biosynthesis	Chalcone and stilbene synthase family protein	0.00024	Up
	Aldo/keto reductase family oxidoreductase	0.00084	Up
	Isoflavone 2’-hydroxylase	0.00144	Up
	Isoflavone 4’-*O-*methyltransferase	0.00212	Up
	Caffeoyl-CoA 3-*O-*methyltransferase	0.01600	Down
	Chalcone-flavonone isomerase family protein	0.01774	Up
	Cytochrome P450 family cinnamate 4-hydroxylase	0.04752	Up

Subsequently, alfalfa roots producted antioxidant molecules in response to oxidative stress caused by OCA. This experiment revealed that OCA induced change in the glycolysis/gluconeogenesis pathway in alfalfa roots. Furthermore, it was observed that NADPH played a role in detoxifying abiotic factors by working in tandem with the cytochrome P450 monoxygenating enzymatic hydrolysis system [12]. NADPH may safeguard hemoglobin and vital sulfhydryl groups against oxidation via reduced glutathione [13]. During the process of gluconeogenesis, uncomplicated non-sugar precursors (lactose, glycerol, glycogenic amino acids, etc.) can be transmuted into glucose or glycogen, which can serve as basic materials for glycolytic reaction [14]. Plant glycolysis and gluconeogenesis processes interplay to enhance and repress each other, providing adequate energy for plants [15].

In the glutathione metabolic pathway, alfalfa roots were treated with OCA. This resulted in a down-regulation of the glutamate-cysteine ligase catalytic subunit and gamma-glutamyl transpeptidase/glutathione hydrolase, along with an up-regulation of glutathione *S-*transferase. These changes prevent glutathione hydrolysis and facilitate glutathione binding reaction, ultimately protecting biomolecules such as DNA and proteins from oxidative damage and completing the detoxification of exogenous substances (Table 2).

Glutathione is a prevalent loe-molecular-weight antioxidant present in plants. Its abundance is closely associated with plant tolerance towards biotic and abiotic stresses. Maintenance of optimal glutathione levels is critical to maintain a stable antioxidant environment and regulate redox-sensitive signals within tissues [16]. Glutathione *S*- transferase is a multifunctional enzyme that plays a crucial role in the in glutathione metabolism pathway of plants. It is a member of the superfamily of enzymes and is primarily associated with the primary and secondary metabolism of plants, as well as their detoxification process [17]. This enzyme can transport and excrete toxic substances in cells, protect plants from surface oxidative damage on their surfaces, isolate heterologous substances, and repair damage to plants [18]. In this study, the up-regulation of glutathione *S*- transferase expression with OCA treatment suggested the vital role of the glutathione metabolic pathway in protecting alfalfa roots against OCA infection. Furthermore, the mutation rax1-1 of GSH1 in Arabidopsis leads to changes in the GSH metabolic process, indicating a possible relationship between GSH metabolism, signal transduction, and expression control of defense genes [19]. Currently, augmenting the expression of glutathione *S-* transferase is deemed to be a crucial indicator of plant response to stress.

Phenylalanine ammonia lyase, *trans*-cinnamic acid 4-monooxygenase, 4-coumaric acid-CoA ligase and caffeic acid 3-*O-*methyltransferase were up-regulated in the biosynthesis pathway of phenylpropanes. The phenylpropane enzyme system could serve as a physiological indicator of plant disease resistance, particularly phenylalanine lyase (PAL) which is known to increase significantly in response to biotic and abiotic stress. Phenylpropane metabolites act as chemical barriers to disease resistance, and the resistance of plants to foreign substances must have a physical groundwork. Phenylpropane metabolites, including phenolic, isoflavone phytochemicals, and lignin, play a crucial chemical barrier role in plant disease resistance (Table 2).

The biosynthesis pathway of phenylpropanoids in alfalfa roots undergoes significant effects from OCA treatment, and the enzyme activity of phenylalanine lyase was up-regulated. This indicated an actives role of phenylpropanoid metabolism in alfalfa roots in responding to OCA-related stress. The biosynthetic pathway of phenylpropanes in plants is subject to influences from biological, abiotic, and pathogenic factors. Recent research suggests that the phenylpropane biosynthetic pathway plays a vital role in the secondary metabolic pathways of plant disease resistance, producing lignin, phytochemicals and other compounds [20]. Phenylalanine ammonia lyase, a key enzyme in the phenylpropane biosynthesis pathway, is likewise associated with plant disease resistance [21]. Studies demonstrated that infected plants produce higher levels of phenylalanine ammonia lyase activity compared to healthy plants. Flavonoids are phytochemicals that result from phenylpropane metabolism following plant infection. The pace of their production and accumulation of substances is linked to the resistance to disease [22]. Numerous studies have demonstrated that pathogen infection induces changes in PAL activity within plants. In particular, PAL activity has been found to increase after infection, exhibiting a time-dependent pattern of ‘initial slow rise, followed by a sharp increase to peak, and then a rapid declin’ [23]. Given these findings, it can be inferred that changes in PAL activity closely correlate with disease resistance.

Chalcone synthase and *trans*-cinnamic acid 4-monooxygenase were up-regulated in the flavonoid biosynthesis pathway. Flavonoids constitute one of the three primary plant protection elements, exerting strong antioxidant effects and delaying cell aging. Additionally, flavonoid accumulation may cause stunted plant growth. In this experiment, the short alfalfa roots might be attributed to flavonoid accumulation under the influence of OCA (Table 2).

Flavonoids are secondary metabolites, belonging to the class of polyphenols and possessing either a C6-C3-C6 or phenylbenzopyran structure [24]. Experimental findings have evidenced their potent antioxidant activity [25]. The decrease of flavonoid content is related to free radical production and membrane lipid peroxidation. The reduction in flavonoid concentration correlates with the generation correlates with the peroxidation of membrane lipids. Ravera’s study demonstrates that rutin (S1F Fig), a flavonoid, can reduce free radical production and membrane lipid peroxidation in tartary buckwheat, ultimately mitigating the harm caused by glyphosate on the plant [26]. In this study, the up-regulation of chalcone synthase and *trans*-cinnamic acid 4-monooxygenase, which were related to flavonoid metabolism, were observed in alfalfa roots that were stressed by OCA. This suggests that the flavonoids in alfalfa roots underwent changes as a response to external abiotic stress. Additionally, the root length was noticeably restricted and the number of lateral was exposed to OCA. The build-up of flavonoids may result in stunted growth of plants, but more research is required to determine the exact mechanism.

### Qualitative and quantitative analysis of different flavonoid metabolites

**Fig 4:**
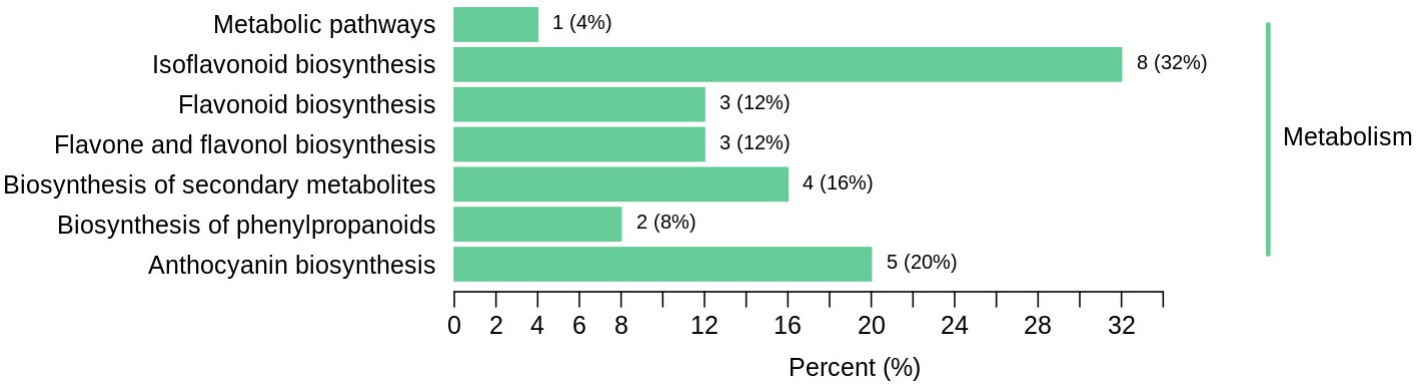
KEGG analysis of differential metabolites.

The enrichment analysis of DAPs demonstrated that proteins were predominantly enriched in the biosynthetic pathway of flavonoids, which were associated with the size of plant roots. Subsequently, experiments were conducted to meticulously qualitatively and quantitatively analyze flavonoids in alfalfa roots. Flavonoid metabolites in alfalfa roots treated with OCA were analysed using UPLC-ESI-MS/MS technology and database. In this study, 87 different flavonoid metabolites were identified, comprising of 14 isoflavones, 2 flavanols, 16 flavonols, 32 flavones, 9 anthocyanins, 10 dihydroflavones, 1 dihydroflavonol and 4 chalcones. Six metabolites, notably paeoniflorin and glabridin, were found to have increased expression, while others including kaempferol 3-*O-*(6’-*O-*acetyl) glucoside, malonyl daidzein, ononin and baicalin found to have decreased expression (Table 3). The up-regulated substances exhibit antioxidative and free radical scavenging properties, which protect alfalfa roots from the toxic effects of OCA and minimize oxidative damage [27–35]. The differential metabolites were categorised into 7 biological processes associated with KEGG analysis with a high level of significance (*P* < 0.05). These processes include ‘anthocyanin biosynthesis’, ‘biosynthesis of secondary metabolites’, and ‘flavone and flavonol biosynthesis’ (Fig 4).

**Table 3:** Qualitative and quantitative analysis of different metabolites of flavonoids. VIP stands for Variable importance projection, *Log FC* stands for log fold change.

Name	Class II	VIP	Fold-Change	Log FC	Type
Peonidin	Anthocyanins	1.3570	2694.0000	11.3960	Up-regulation
Glabranine	Dihydroflavone	1.3310	1930.5560	10.9130	Up-regulation
Wighteone	Isoflavones	1.3140	1641.0000	10.6800	Up-regulation
Quercetin-3-*O*-*β-D-*xylopyranoside	Flavonols	1.2130	539.8630	9.0760	Up-regulation
Avicularin	Flavonols	1.1860	500.2740	8.9670	Up-regulation
Scutellarin	Flavonoid	1.0870	164.3260	7.3600	Up-regulation
2,6-Dimethy l-7-octene-2,3,6-triol	Flavonoid	1.0670	0.0327	-4.9330	Down-regulation
Apigenin 7-rutinoside (Isorhoifolin)	Flavonoid	1.0650	0.0274	-5.1920	Down-regulation
Apigenin 5-*O-*glucoside	Flavonoid	1.0450	0.0145	-6.1040	Down-regulation
Chrysoeriol-*O-*glucuronic acid	Flavonoid	1.0300	0.0080	-6.9190	Down-regulation
Daidzein-4’-glucoside	Isoflavones	1.1610	0.0060	-7.3040	Down-regulation
Eriodictyol 7-*O-*glucoside	Dihydroflavone	1.0910	0.0060	-7.4040	Down-regulation
Luteolin-8-*C-*hexosyl-*O-*hexoside	Flavonoid	1.0590	0.0050	-7.5550	Down-regulation
Isorhamnetin-*O-*rutinoside	Flavonols	1.1360	0.0040	-7.9500	Down-regulation
Eriodictyol *C-*hexoside	Dihydroflavone	1.1570	0.0030	-8.1700	Down-regulation
Peonidin *O-*hexoside	Anthocyanins	1.1410	0.0030	-8.2270	Down-regulation
Peonidin 3-*O-*glucoside	Anthocyanins	1.1640	0.0030	-8.4360	Down-regulation
Tricin 7-*O-*hexosyl-*O-*hexoside	Flavonoid	1.1640	0.0030	-8.6020	Down-regulation
5,2’-Dihydroxy-7,8-dimethoxyflavone glycosides	Flavonoid	1.1850	0.0020	-8.6650	Down-regulation
Quercetin-5-*O-*malonylhexosyl-hexoside	Flavonols	1.1770	0.0020	-8.6850	Down-regulation
Apigenin-7-*O-β-D-*glucuronide	Flavonoid	1.3920	0.0020	-8.8390	Down-regulation
Malvidin 3-*O-*glucoside (Oenin)	Anthocyanins	1.2120	0.0020	-9.1780	Down-regulation
Lonicerin	Flavonoid	1.2050	0.0010	-9.5330	Down-regulation
5,7-Dihydroxy-3’,4’,5’-trimethoxyflavone	Flavonoid	1.249	0.0010	-9.6800	Down-regulation
Isoformononetin	Isoflavones	1.2520	0.0010	-9.7610	Down-regulation
Isobavachalcone A	Chalcones	1.2610	0.0010	-9.7880	Down-regulation
Kaempferol-3,7-*O-*diglucoside	Flavonols	1.2450	0.0010	-9.8800	Down-regulation
Quercetin-7-*O-*malonylhexosyl-hexoside	Flavonols	1.2720	0.0010	-9.9940	Down-regulation
Malvidin 3-*O-*galactoside (Primulin)	Anthocyanins	1.2760	0.0010	-10.0240	Down-regulation
Farrerol 7-*O-*glucoside	Dihydroflavone	1.2850	0.0009	-10.1750	Down-regulation
Isorhamnetin-*O-*Hexoside-*O-*Hexoside	Flavonols	1.2330	0.0008	-10.2070	Down-regulation
Petunidin 3-*O-*glucoside	Anthocyanins	1.2660	0.0008	-10.2550	Down-regulation
Isorhamnetin-*O-*Hexoside-*O-*acetyl-*O-*Hexoside	Flavonols	1.2900	0.0008	-10.2580	Down-regulation
Cyanidin 3-*O-*galactoside	Anthocyanins	1.2710	0.0008	-10.3780	Down-regulation
Isochrysoeriol *O-*dihexoside	Flavonoid	1.2770	0.0007	-10.4480	Down-regulation
Tricin 7-*O-*Glucuronide	Flavonoid	1.2980	0.0007	-10.5300	Down-regulation
Isobavachalcone B	Chalcones	1.3070	0.0007	-10.5540	Down-regulation
3’,4’,7-Trihydroxyflavone	Flavonoid	1.3060	0.0007	-10.5620	Down-regulation
Epiafzelechin	Flavanols	1.3130	0.0006	-10.6320	Down-regulation
Echinatin	Chalcones	1.3170	0.0006	-10.7520	Down-regulation
Isorhamnetin-*O-*Hexoside-*O-*malonyl-*O-*Hexoside	Flavonols	1.2920	0.0006	-10.8200	Down-regulation
Gallocatechin-Gallocatechin	Flavanols	1.3010	0.0004	-11.2560	Down-regulation
Pelargonidin 3-*O-*glucoside	Anthocyanins	1.3500	0.0004	-11.3250	Down-regulation
Baicalein	Flavonoid	1.3640	0.0004	-11.4570	Down-regulation
Chrysoeriol-*O-*malonylhexoside	Flavonoid	1.3640	0.0003	-11.5120	Down-regulation
Pinocembrin(Dihydrochrysin)	Dihydroflavone	1.3520	0.0003	-11.5700	Down-regulation
Apigenin-3-*O-α-L-*rhamnoside	Flavonoid	1.3750	0.0003	-11.6690	Down-regulation
Liquiritigenin-7,4-diglucoside	Dihydroflavone	1.3790	0.0003	-11.8820	Down-regulation
Apigenin-7-*O-*(6-*O-*Malonyl Glucoside)	Flavonoid	1.3772	0.0003	-11.9520	Down-regulation
Tricin *O-*saccharic acid	Flavonoid	1.4003	0.0002	-12.1250	Down-regulation
Luteolin-7-*O-*glucoside(Cynaroside)	Flavonoid	1.4023	0.0002	-12.1260	Down-regulation
Hesperetin *O-*Glucuronic acid	Dihydroflavonol	1.4035	0.0002	-12.1980	Down-regulation
Malonylglycitin	Isoflavones	1.4155	0.0002	-12.3660	Down-regulation
Kaempferol 3-*O-*(6’-malonyl)galactoside	Flavonols	1.4043	0.0002	-12.4540	Down-regulation
Kaempferol-7-*O-*glucosdie	Flavonols	1.4226	0.0002	-12.4730	Down-regulation
Isorhamnetin-hexose-malonate	Flavonols	1.0020	0.0002	-12.5250	Down-regulation
Calycosin-7-glucoside	Isoflavones	1.4342	0.0002	-12.7010	Down-regulation
3’-Methoxydaidzin	Isoflavones	1.4316	0.0001	-12.7940	Down-regulation
Sissotrin	Isoflavones	1.4337	0.0001	-12.8560	Down-regulation
Naringenin-7-*O-*glucoside	Dihydroflavone	1.4385	0.0001	-12.8640	Down-regulation
Luteolin-3’-*O-β-D-*glucoside	Flavonoid	1.4445	0.0001	-12.8770	Down-regulation
Luteolin-di-*O-*glucoside	Flavonoid	1.4463	0.0001	-12.9050	Down-regulation
Kaempferol-3-*O-*glucoside (Astragalin)	Flavonols	1.4529	0.0001	-13.0360	Down-regulation
Acacetin-7-*O-*galactoside	Flavonoid	1.4425	0.0001	-13.0370	Down-regulation
Apigenin-7-*O-*[*β-D-*glucuronopyranosyl(1→2)-*O-β-D-*glucuronopyranoside)	Flavonoid	1.4548	0.0001	-13.0540	Down-regulation
Luteolin-7-*O-β-D-*gentiobioside	Flavonoid	1.45828	0.0001	-13.1110	Down-regulation
6’’-*O-*Acetylgenistin	Isoflavones	1.4407	0.0001	-13.1570	Down-regulation
Quercetin-3-*O-*(2’’-acetyl)-*β-D-*glucuronide	Flavonols	1.4315	0.0001	-13.1710	Down-regulation
Luteolin-4’-*O-β-D*-glucoside	Flavonoid	1.4626	0.0001	-13.1750	Down-regulation
Apigenin-7-*O-*(6’-*O-*acetyl)-*β-D-*glucoside	Flavonoid	1.4502	0.0001	-13.3510	Down-regulation
Chrysoeriol-7-*O-*[*β-D-*glucuronopyranosyl-(1→2)-*O-β-*D-glucuronopyranoside]	Flavonoid	1.4457	0.0001	-13.3520	Down-regulation
Liquiritin	Dihydroflavone	1.4735	0.0001	-13.4150	Down-regulation
Isoliquiritin	Chalcones	1.4645	0.0001	-13.4230	Down-regulation
Apigenin-*O-*Malonyl-*O-*Hexoside-*O-*Pentoside	Flavonoid	1.4708	0.0001	-13.6540	Down-regulation
Tricin 5-*O-*Glucoside	Flavonoid	1.4968	0.0001	-13.8350	Down-regulation
Wistin (6,4’-Dimethoxyisoflavone-7-glucoside)	Isoflavones	1.5020	0.0001	-13.9080	Down-regulation
Isorhamnetin-*O-*acetyl-hexoside	Flavonols	1.4840	0.0001	-13.9140	Down-regulation
Apigenin 7-*O-*glucoside(Cosmosiin)	Flavonoid	1.5339	0.00004	-14.5590	Down-regulation
Malonyglygenistin	Isoflavones	1.5452	0.00004	-14.7370	Down-regulation
Daidzein	Isoflavones	1.5507	0.00003	-14.8300	Down-regulation
Liquiritigenin	Dihydroflavone	1.5605	0.00003	-15.0080	Down-regulation
Genistin(Genistein 7-*O-*Glucoside)	Isoflavones	1.5761	0.00002	-15.3070	Down-regulation
Baicalin	Flavonoid	1.6059	0.00002	-15.8980	Down-regulation
Formononetin 7-*O-*glucoside(Ononin)	Isoflavones	1.6242	0.00001	-16.2520	Down-regulation
Malonyldaidzin	Isoflavones	1.6662	0.000007	-17.1000	Down-regulation
Kaempferol-3-*O-*(6’’-acetyl)-glucoside	Flavonols	1.6961	0.000005	-17.7200	Down-regulation

### Metabolic pathways of flavonoids

**Fig 5:**
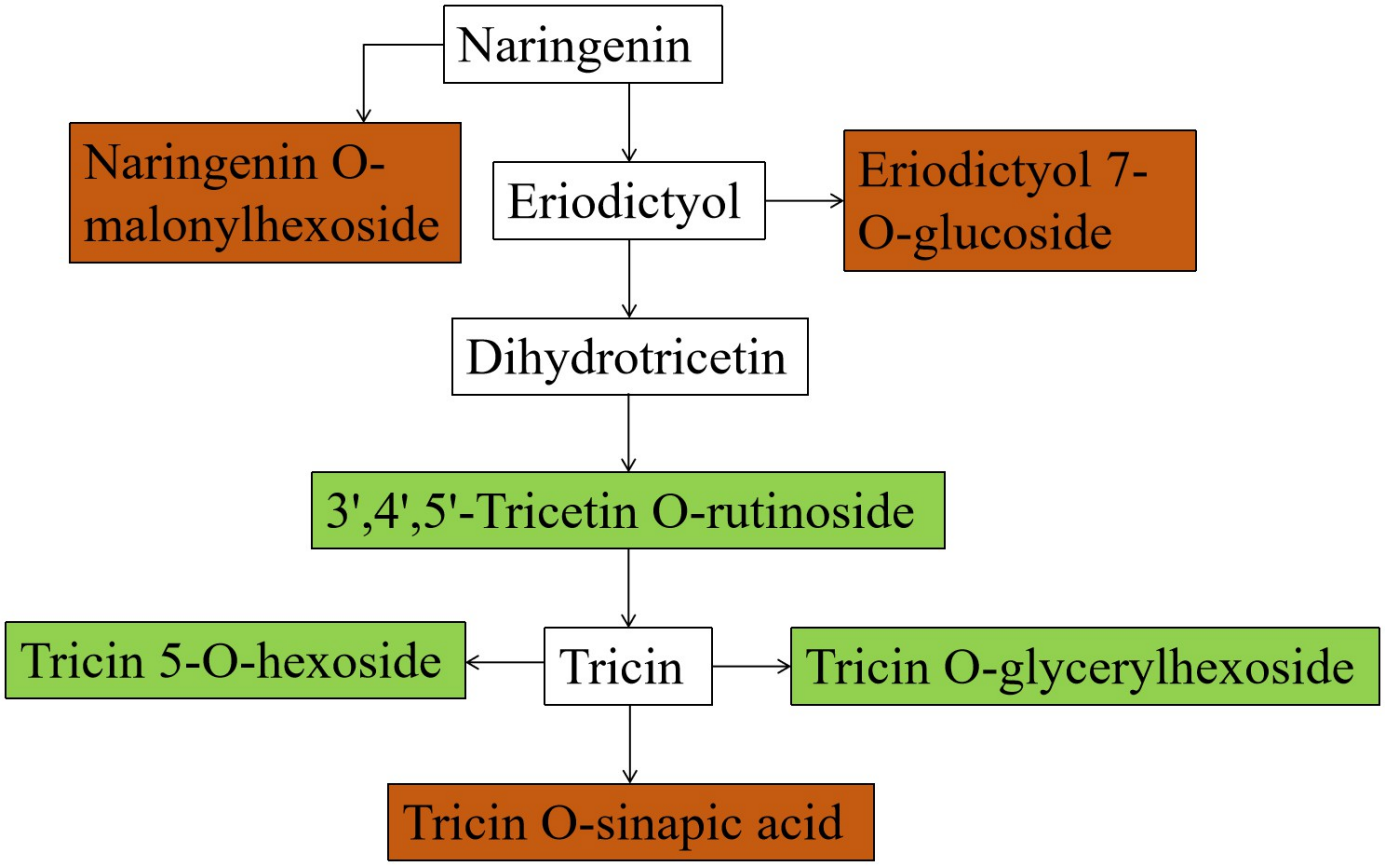
Metabolic pathways of flavonoids. Red indicates that the metabolite content is significantly increased, and green indicates that the metabolite content is significantly decreased.

Differential metabolites interact within organisms and create distinct pathways. These metabolitesare annotated and analysed by the KEGG database [36]. Eriodictyol 7-*O-*glucoside was upregulated, leading to direct participation in eriodictyol generation. The up-regulation of naringenin *O-*malonyhexosides directly influenced naringenin production. The downregulation of 3’, 4’, 5’-trcetin *O-*rutinoside and selgin *O-*malonylhexosine had a direct impact on dihydrotricetion formation. The down-regulation of trcin 5-*O-*hexosine and tricin *O-*glycylhexosine, and up-regulation of tricin *O-*sinapic acid were related to the formation of tricin. Trcin 5-*O*-hexosine, tricin *O*-glycylhexosine and tricin *O*-sinapic acid were found to interact closely with each other and participate in the biosynthesis of naringenin chalcone, thus regulating the growth of alfalfa roots. The interaction between the metabolites led to the regulation process (Fig 5). The alfalfa roots can act to boost the immune system of plants and increase its resistance to OCA stress by impacting the production of flavonoids. The Arabidopsis mutants deficient in flavonoids have increased endogenous ROS levels and heightened sensitivity to ROS-generating stresses, along with reduced antioxidant capacity [37]. Naringenin chalcone (NAC) can be synthesised into dihydroflavonoid naringenin and then into flavonoid apigenin, with the ability to scavenge free radicals through a chain reaction involving including propagation and termination steps thereby impacting the biological antioxidant process [38].

Proteomic analysis indicated that PAL constitutes the key regulatory protein responsible for secondary substance biosynthesis, including lignin and flavonoids, and is linked to the plant’s disease resistance. Metabolomics analyses demonstrated that OCA primarily impacted the synthesis of naringenin chalcone in the root system of alfalfa. The investigation displayed various classes of flavonoids, the chalconoid naringenin chalcone, the flavanones naringenin and dihydrotricin, and the flavone tricin, incorporated into the lignin polymer of papyrus (*Cyperus papyrus* L.) rind [39]. PAL impacts lignin formation in the alfalfa root system through the regulation of lignin production and the biosynthesis of naringenin chalcone. This, in turn, affects cell wall formation, leading to the destruction of the root system structure, ultimately resulting in wilting and death of alfalfa. All of these findings suggested that OCA caused harm to the cell walls in the alfalfa roots, which ultimately led to the decline in their growth.

## Conclusions

There were 680 DAPs with OCA treatment. Among them, 333 proteins were up-regulated and 347 proteins were down-regulated. Enrichment analysis determined that these DAPs played a significant role in various molecular, functional and biological processes, particularly in ribosome, phenylpropanoid biosynthesis, glutathione metabolism, glycolysis/gluconeogenesis and flavonoid biosynthesis. In addition, phenylalanine deaminase was identified as a potential chemical-induced regulation target associated with plant lignin formation. DAPs were mainly enriched in flavonoid biosynthesis pathways, which were related to plant root size. Using the UPLC-ESI-MS/MS technique and database, a total of 87 flavonoid metabolites were discovered. The metabolites were predominantly enriched for biosynthesizing naringenin chalcone, which was linked to plant lignin formation, aligning with the enrichment outcomes of DAPs.

## Supporting information

S1 Fig Structure of chemical substances. (TIF)

S2 Fig The differentially accumulated proteins under OCA stress. (TIF)
