# Supplementary figures and images for: Quantitative proteomics and metabolomics analysis reveals the response mechanism of alfalfa (Medicago sativa L.) to o-coumaric acid stress
Source: PLoS One. 2023 Dec 8;18(12):e0295592. doi: 10.1371/journal.pone.0295592 (PMC10707586; doi:10.1371/journal.pone.0295592)

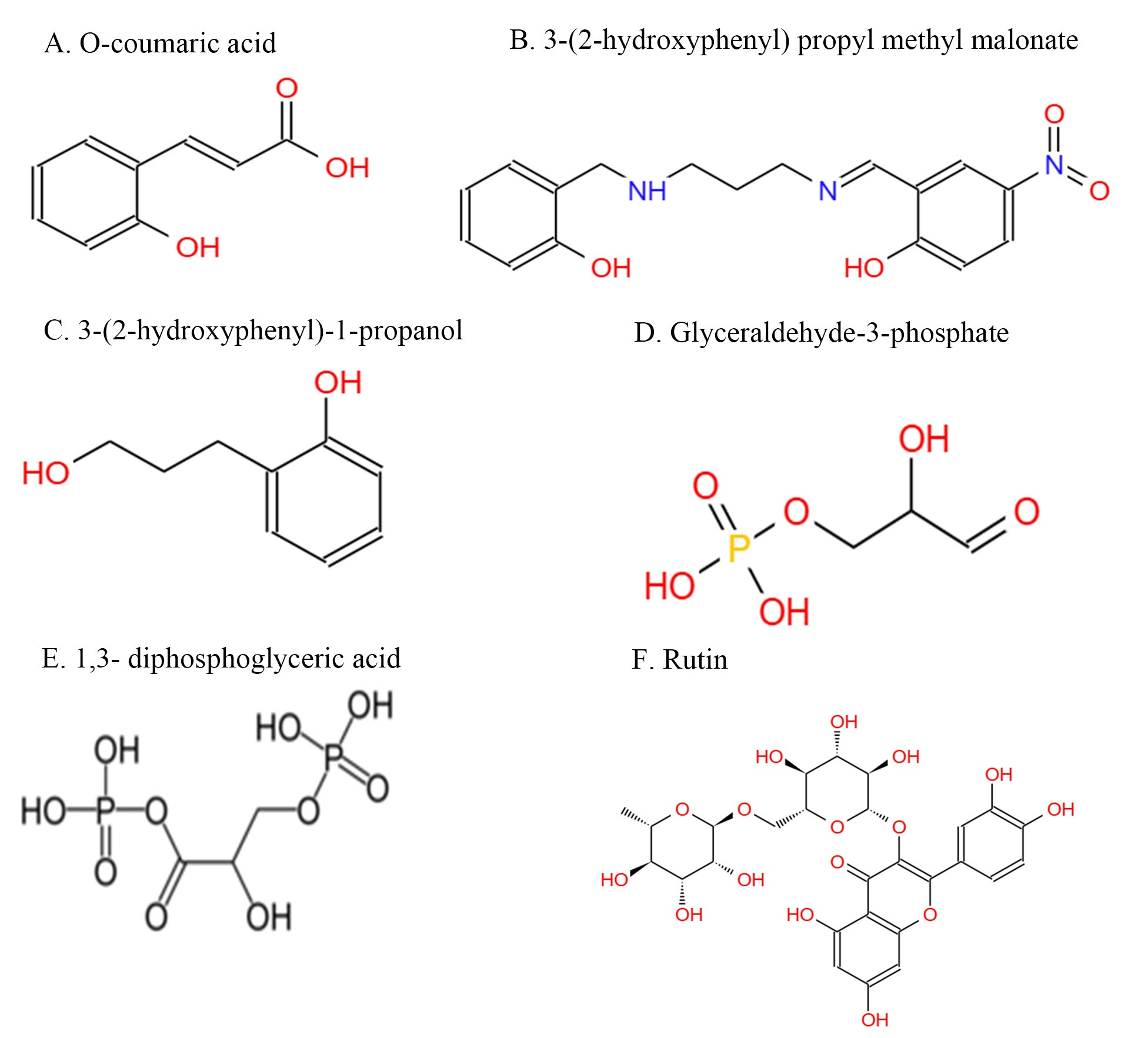

Supplement: S1 Fig — (TIF) [file pone.0295592.s001.tif]

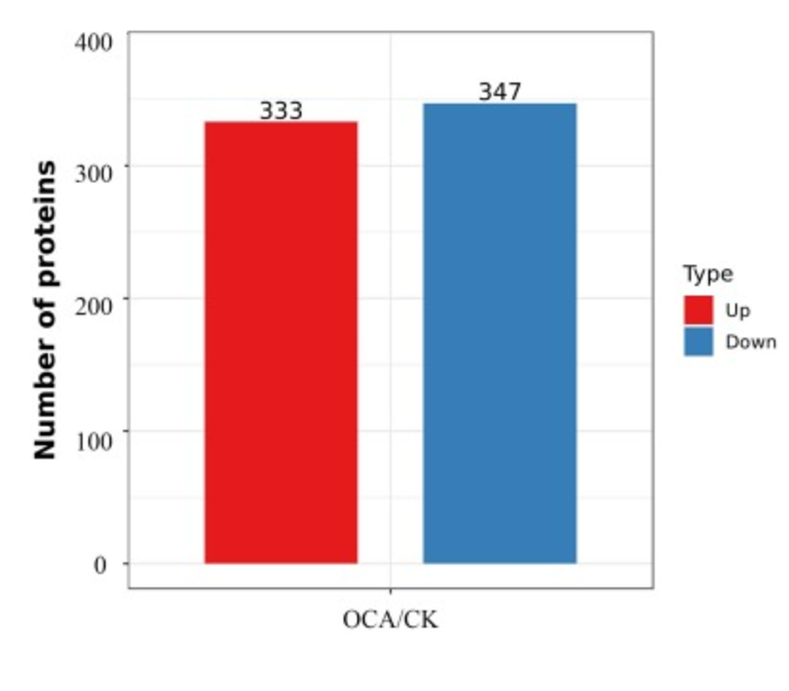

Supplement: S2 Fig — (TIF) [file pone.0295592.s002.tif]
